# YouTube Videos and Informed Decision-Making About COVID-19 Vaccination: Successive Sampling Study

**DOI:** 10.2196/28352

**Published:** 2021-05-06

**Authors:** Charles E Basch, Corey H Basch, Grace C Hillyer, Zoe C Meleo-Erwin, Emily A Zagnit

**Affiliations:** 1 Teachers College, Columbia University New York, NY United States; 2 William Paterson University Wayne, NJ United States; 3 Mailman School of Public Health, Columbia University New York, NY United States; 4 Milken Institute School of Public Health, George Washington University Washington, DC United States

**Keywords:** YouTube, vaccination, COVID-19, social media, communication, misinformation, disinformation, adverse reactions

## Abstract

**Background:**

Social media platforms such as YouTube are used by many people to seek and share health-related information that may influence their decision-making about COVID-19 vaccination.

**Objective:**

The purpose of this study was to improve the understanding about the sources and content of widely viewed YouTube videos on COVID-19 vaccination.

**Methods:**

Using the keywords “coronavirus vaccination,” we searched for relevant YouTube videos, sorted them by view count, and selected two successive samples (with replacement) of the 100 most widely viewed videos in July and December 2020, respectively. Content related to COVID-19 vaccines were coded by two observers, and inter-rater reliability was demonstrated.

**Results:**

The videos observed in this study were viewed over 55 million times cumulatively. The number of videos that addressed fear increased from 6 in July to 20 in December 2020, and the cumulative views correspondingly increased from 2.6% (1,449,915 views) to 16.6% (9,553,368 views). There was also a large increase in the number of videos and cumulative views with respect to concerns about vaccine effectiveness, from 6 videos with approximately 6 million views in July to 25 videos with over 12 million views in December 2020. The number of videos and total cumulative views covering adverse reactions almost tripled, from 11 videos with approximately 6.5 million (11.7% of cumulative views) in July to 31 videos with almost 15.7 million views (27.2% of cumulative views) in December 2020.

**Conclusions:**

Our data show the potentially inaccurate and negative influence social media can have on population-wide vaccine uptake, which should be urgently addressed by agencies of the United States Public Health Service as well as its global counterparts.

## Introduction

At the end of 2019, the World Health Organization (WHO) was informed by the Chinese health authorities about a cluster of pneumonia cases, which was shortly thereafter attributed to a novel coronavirus (SARS-CoV-2) [1]. By the end of January 2020, the WHO characterized these outbreaks as a public health emergency [2]. At the time of writing this manuscript, approximately 1 year following this declaration, over 2 million deaths worldwide [3] had been directly attributed to COVID-19, the disease caused by SARS-CoV-2. At a similar time, the United States’ Centers for Disease Control and Prevention (CDC) issued a warning that a variant of SARS-CoV-2, first identified in England in late 2020 and known as “B.1.1.7,” had been detected in at least 10 US states [4]. Research suggests that B.1.1.7, as with other identified variants circulating globally, is more highly transmissible. The spread of the variant is of great public health concern in terms of repercussions on case counts and, consequently, hospital capacity and eventual mortality [4]. To say that the impacts of the COVID-19 global pandemic on morbidity, mortality, and global economies have been devastating would be a vast understatement. The degree to which the pandemic has exacerbated preexisting economic and health inequities has been staggering. Yet, the record speed with which multiple COVID-19 vaccinations were developed and received emergency use authorization in under a year’s time not only provides hope but represents an astounding scientific accomplishment.

In early January 2020, scientists first made the genome sequence of SARS-CoV-2 available on the web [5], and by mid-March 2020, Moderna’s experimental messenger RNA (mRNA)-based vaccine entered phase 1 of clinical trials [6]. By early December 2020, regulators in the United Kingdom approved the emergency authorization status for the Pfizer and BioNTech COVID-19 vaccines [7], and 6 days later, the United States Food and Drug Administration (FDA) followed suit [8]. By December 18, 2020, a second mRNA vaccine developed by Moderna was also granted emergency use authorization in the United States by the FDA [9]. Although the rollout of vaccines in many countries has been slower than anticipated [10], as of March 1, 2021, nearly 7.8 billion vaccinations have been administered globally [11]. It is well known that the pipeline from vaccine development to distribution is normally rather slow, in no small part due to the tremendous expense involved. However, the rapid sequencing of the virus (SARS-CoV-2), international scientific collaboration, and government financial support [6] have helped to dramatically speed up the pace in this case. The nature of the mRNA vaccines, which do not require culturing or fermentation but instead rely on synthetic RNA, has further facilitated more rapid development [6].

Despite the highly encouraging safety and efficacy profiles of COVID-19 vaccines that have been granted emergency use authorization, thus far, the very processes that allowed for rapid development have also been a source of public concern, with possible negative effects on the uptake of vaccination [12]. Vaccine hesitancy is multi-factorial phenomenon, often driven by a confluence of factors. Not the least of which is a mistrust of scientific experts and government officials [13], which, for some populations, is grounded in the trauma of racist exploitation, disregard, and injustice [14]. Although vaccine hesitancy has a long history [15,16], it is fair to say that today, the internet facilitates, if not drives, both vaccine misinformation and disinformation [17]. Vaccine misinformation pertains to erroneous conclusions based on incomplete or incorrect facts, whereas vaccine disinformation involves the purposeful spread of falsehoods related to both specific vaccines and vaccination, in general [17]. The spread of misinformation is likely facilitated by fear and misunderstanding of vaccine development and approval processes. In contrast, in the latter case, the intent is clearly nefarious in nature.

Social media platforms have become a dominant communication channel through which people seek and share health-related information [18,19]. Research suggests that this is no less the case for information on COVID-19 [20]. Although different age cohorts tend to prefer different social media platforms, overall, YouTube is extremely popular, with nearly three-quarters of the US adult population known to have used the platform [21]. Founded in 2005, YouTube has over 2 billion users [22]. YouTube videos can be accessed in 80 different languages, and over a billion hours of video are streamed every day [22]. As with social media platforms in general, health-related content shared on YouTube is often not empirically grounded and yet can easily be accessed [23]. Research on coronavirus-related videos on YouTube is nascent but the results thus far are mixed, with some studies finding that the majority of video content is reliable [24,25], whereas other studies, including those previously undertaken by authors of this study group [26], demonstrating otherwise. At the time of this study, there is little published research on COVID-19 vaccination content on YouTube, with the exception of our previous study that revealed that the majority of videos were uploaded by news outlets and did not contain misinformation [27]. Our prior investigation was conducted relatively early in the pandemic (early-April 2020). Continued monitoring and analysis of social media coverage of COVID-19 vaccine messages is vital to improve its understanding among public health officials about responding to questions and concerns that may produce vaccine hesitance and impede community mitigation. The purpose of this study was, therefore, to build on and update the findings of our previous investigation and add to the repository of scientific knowledge on COVID-19 social media content.

## Methods

Using a cleared browsing history, and the keywords “coronavirus vaccination,” we searched YouTube for relevant videos, sorted them by view count, and conducted a successive sampling study. Two successive samples (with replacement) were selected and each included the 100 most widely viewed videos in July and December 2020, respectively. Half of the videos in each sample were independently coded by one researcher (EZ or CHB), and a 10% random sample was coded by both researchers to demonstrate inter-rater reliability (using the Kappa coefficient), which was found to be high (κ=.969 in Round 1 and κ=.963 in Round 2). Metadata were gathered for each video, including date uploaded, source, length (in minutes), and number of views. A video content checklist developed for this and our prior study of vaccine use on YouTube was based on a CDC fact sheet [27]. Content coverage related to vaccine development, fast-tracking, emergency use authorization, manufacturing, dissemination, eligibility, dosing, herd immunity, concerns about adverse reactions, fear, effectiveness, and immunity duration were dichotomously coded as “present” or “absent.” The analysis comprised frequency and percentage distributions for dichotomous content variables and the proportion of total cumulative views garnered by videos addressing each content category. For continuous variables (number of video views and length of video), mean and SD were computed. Analysis was conducted within each of the successive samples using SPSS software (version 25.0; IBM Corp.). At William Paterson University and Columbia University, studies that do not involve human subjects are not subject to review; the Institutional Review Board at Teachers College of Columbia University reviewed the study protocol and deemed the research exempt from review.

## Results

The videos evaluated in this study were viewed over 55 million times. Twenty-nine of the videos from the July sample were retained in December. The mean length of the videos in the two samples was 7.5 minutes (see Table 1). Over 80% of the widely viewed videos in each sample originated from television or internet news, whereas fewer than 10% of the videos originated from consumers, professionals, or entertainment television. Between the two rounds, there were 14 professional videos, 7 in each round with 4 overlapping between the rounds. The professional videos in Round 1 comprised 4.4% of the total views (2,403,245/55,086,261) and those in Round 2 comprised 3.8% of the total views (2,157,142/57,506,506).

The vaccine development process was the most covered topic, followed by fast-tracking of the vaccine (see Table 2). The vaccine manufacturing process was covered in 31 videos in July 2020 and 36 videos in December 2020, garnering almost one-third of the cumulative views in each sample. There was a 44% increase in the share of cumulative views of videos addressing vaccine dissemination from July (10,197,203/55,086,261, 18.5%) to December 2020 (14,732,085/57,506,506, 25.6%). This is attributable to the approximately 60% increase in videos covering this topic, from 17 in July 2020 to 27 in December 2020. From July to December, videos covering vaccine eligibility more than doubled (from 12 to 25), with the cumulative views increasing from <5.5 million to >9.5 million; however, even in December 2020, videos covering this topic accounted for only 16.8% (9,652,883/57,506,506) of the cumulative views. The number of videos addressing vaccine dosing increased from 4 to 26, and cumulative views of videos addressing dosing increased from 4% (2,217,251 views) to 15.7% (9,017, 039 views). There was relatively little change in the percentage of cumulative views garnered by videos addressing herd immunity or the duration of immunity derived from COVID-19 vaccines.

**Table 1:** Characteristics of successive samples of YouTube videos about COVID-19 vaccination, July through December 2020.

Characteristic		Value, n (%)	
		July 2020 (n=100)	December 2020 (n=100)
**Video views**			
	Total views	55,086,261	57,506,506
	Mean views (SD)	550,863 (620,691)	575,065 (604,247)
	Range	135,729-4,016,406	196,294-4,038,435
**Video length (minutes)**			
	Mean length (SD)	8.2 (9.4)	7.2 (6.3)
	Range	0.4-51.4	0.5-35.4
**Source**			
	Consumer	5 (5)	8 (8)
	Professional	7 (7)	7 (7)
	Television or internet news	84 (84)	81 (81)
	Entertainment television	4 (4)	4 (4)

**Table 2:** Content of successive samples of YouTube videos about COVID-19 vaccination, July through December 2020.

Content covered	July 2020				December 2020		
	Number of videos (n)	Number of views (n)	Percentage of cumulative views (%)	Number of videos (n)		Number of views (n)	Percentage of cumulative views (%)
Vaccine development	77	47,745,687	86.7	93		52,907,010	92.0
Fast-tracking	57	31,891,480	57.9	70		40,140,849	69.8
Emergency use authorization	3	593,609	1.1	22		10,132,084	17.6
Vaccine manufacturing	31	17,498,885	31.8	36		18,817,111	32.7
Vaccine dissemination	17	10,197,203	18.5	27		14,732,085	25.6
Vaccine eligibility	12	5,410,203	9.8	25		9,652,883	16.8
Vaccine dosing	4	2,217,251	4.0	26		9,017,039	15.7
Herd immunity	5	2,286,901	4.2	6		3,173,062	5.5
Adverse reactions to the vaccine	11	6,456,465	11.7	31		15,686,832	27.3
Fear	6	1,449,915	2.6	20		9,553,368	16.6
Concerns about effectiveness	6	5,966,961	10.8	25		12,317,526	21.4
Concerns about immunity duration	5	2,415,092	4.4	10		3,953,045	6.9

In contrast, the number of videos that addressed fear increased from 6 (July 2020) to 20 (December 2020), and the corresponding percentage of cumulative views increased from 2.6% (1,449,915 views) to 16.6% (9,553,368 views). There was also a large increase in the number of videos and cumulative views between the two samples with respect to concerns about vaccine effectiveness, from 6 videos with approximately 6 million views to 25 videos with over 12 million views, and a commensurate increase in the proportion of cumulative views (from 10.8% to 21.4%). The number of videos and total cumulative views covering adverse reactions almost tripled from 11 videos with approximately 6.5 million views (11.7% of cumulative views) in July to 31 videos with almost 15.7 million views (27.2% of cumulative views) in December 2020.

## Discussion

Vaccinations have resulted in the eradication of small pox and considerable reductions in measles, mumps, rubella, polio, varicella, and many other infectious diseases [28]. Studying vaccinations historically shows the large time gaps that occur between scientific conceptualization, development, manufacturing, approval, and population-wide uptake. The current pandemic provides a remarkable example of unprecedented speed in developing, testing, and emergency use authorization of multiple vaccines [6,29,30], and bodes well for primary prevention of COVID-19.

The only two ways to achieve primary prevention of COVID-19 is by decreasing exposure to SARS-CoV-2 and evolving variants and reducing susceptibility through active infection or vaccination (although the efficacy of vaccines is less than 100% and the duration of immunity conferred through active infection or vaccination is equivocal). As long as COVID-19 is spreading through communities, social distancing, mask use, and hand hygiene are the best ways for reducing exposure among susceptible people [31-33]. Manufacturing and distributing vaccines in ways that result in widespread uptake is the key public health strategy for reducing population-wide susceptibility to COVID-19 [34].

Behaviors for reducing exposure and susceptibility both require voluntary decision-making by individuals. Reducing exposure through social distancing, mask use, avoiding crowded or poorly ventilated spaces, and practicing hand hygiene is challenging for many reasons. Not only are there economic pressures for frontline workers to be around others, but because people are inherently social and have been isolated to a greater or lesser degree since the pandemic was declared a global public health emergency by the WHO in January 2020, it is also inevitable that COVID-19 will continue to be transmitted within and among communities. Hence, reducing susceptibility through vaccination provides the greatest long-term hope for primary prevention of COVID-19.

The main public health challenge now is the population-wide vaccine uptake and concomitant herd immunity. Observations in fields ranging from agriculture to technology indicate that something new—in this case, the uptake of a new vaccine—follows predictable patterns of adoption, with some population segments likely to adopt an innovation, and successive population segments adopting at slower rates over time until the last segment—laggards, who are most resistant and may never adopt the innovation [35]. A substantial proportion of the United States [36] and the global [37] population is reportedly hesitant to receive a COVID-19 vaccination. In the United States, population segments that appear most hesitant vary by demographic and social characteristics; for example, those who appear to be more hesitant are women, younger and middle-aged adults, non-Hispanic Black people, adults with lower income and educational attainment and no health insurance, and adults residing in nonmetropolitan areas [36,38]. Various reasons for vaccine hesitancy have been identified, including concerns about side effects, safety, effectiveness, lack of trust in the government, and how politics has influenced vaccine development [35].

The main implication for public health education is that different messages are more or less relevant to assist different population segments to make informed decisions about vaccination, and the nature of messaging is dynamic and influenced by rapidly changing social context. Communication strategies have been proposed based on the level of vaccine hesitancy [39]. The current challenge is different than those of the past, not only because the speed with which new information about COVID-19 and vaccination effectiveness and availability is being generated but also because of the speed with which information is disseminated throughout the population via social media. Although a very small proportion of serious adverse reactions have occurred following the 76+ million doses of COVID-19 vaccinations administered between December 14, 2020, and March 1, 2021 [40], our results show that the number of widely viewed YouTube videos covering the topic of adverse reactions to COVID-19 vaccine almost tripled from 11 in July to 31 in December 2020, with a commensurate increase in the proportion of cumulative views (from 11.7%, representing 6.4 million views, to 27.2%, representing 15.6 million views). The coverage of concerns about effectiveness more than quadrupled with regard to the number of videos (6 videos in July to 25 videos in December 2020), and almost doubled with regard to the proportion of cumulative views (from 10.8%, representing approximately 6 million views, to 21.4%, representing >12 million views). The extent to which messages are widely viewed can affect consumers’ beliefs and decision-making regarding the uptake of COVID-19 vaccination. Public health agencies responsible for helping people make informed decisions about vaccination must, therefore, monitor widely viewed social media on a daily basis to identify and address sources of misinformation and disinformation. In the context of this global public health emergency, we believe social media companies also share this responsibility [41].

A comprehensive national prevention strategy is needed to mitigate further morbidity and mortality caused by COVID-19, and an essential element of this strategy is to discover ways to assist the public in making informed decisions about vaccination [42,43]. Disseminating up-to-date and accurate information through social media is one of the most effective ways to reach a large proportion of the population. To date, public health agencies have had limited effectiveness in achieving this goal. Equally, if not more concerning, is that the efforts by individuals and groups to discourage vaccination are effectively reaching people who are uncertain or ambivalent about being vaccinated [44]. It is also essential for an effective national prevention strategy to recognize and address other barriers that preclude individuals’ ability to make informed decisions about vaccination, such as limited access to the internet necessary to schedule an appointment, loss of income from taking time off from work, and lack of transportation.

This study is delimited in scope in several ways. First, only the time period between July and December 2020 was sampled. The choice of these two points in time was somewhat arbitrary, but represents different pivotal points in the vaccine development process. Second, only 100 videos were included in each sample. Third, only certain content was coded. Fourth, attributes of videos were only examined in relation to the number of views, and we cannot distinguish between the number of views and the number of viewers. We did not have data on the characteristics of viewers such as geography or demographics, nor did we know the extent to which, if any, these videos impacted behavior. Finally, we relied on the keywords “coronavirus vaccine” to search for and sort the videos; thus, we relied on YouTube search algorithms. The main outcome for this study—the number of views—relied on YouTube numbers and sorting algorithms. Despite these delimitations, with the exception of our pilot study [27], we did not identify any published studies examining YouTube videos related to COVID-19 vaccine messages. Although the sample size was small, the videos examined were widely viewed. This study was intended as a stepping-stone to improve understanding about videos that reach a large number of people. This is not only important for reaching the general population with accurate information about vaccinations but also for being aware and responding to disinformation and misinformation that may be disseminated through widely viewed content on social media, and influence the hesitancy of people who are uncertain about receiving a vaccine.

In conclusion, our data show the potentially inaccurate and negative influence social media can have on population-wide vaccine uptake that should be urgently addressed by agencies of the United States Public Health Service as well as its global counterparts. At the time of this study (the second half of 2020), videos uploaded by public health agencies or professionals have had limited presence among the widely viewed YouTube videos that have reached millions of people. Different approaches are needed to understand and address the concerns subgroups of people have about COVID-19 vaccination. Improving the extent to which social media reaches the public with comprehensible, up-to-date, and scientifically accurate information must be a part of a comprehensive national strategy to help people make informed decisions about vaccination.

## Abbreviations

CDC: Centers for Disease Control and Prevention
FDA: Food and Drug Administration
mRNA: messenger RNA
WHO: World Health Organization
